# Exploring orphan GPCRs in neurodegenerative diseases

**DOI:** 10.3389/fphar.2024.1394516

**Published:** 2024-06-04

**Authors:** Devrim Öz-Arslan, Melis Yavuz, Beki Kan

**Affiliations:** ^1^ Department of Biophysics, Acibadem MAA University, School of Medicine, Istanbul, Türkiye; ^2^ Department of Neurosciences, Acibadem MAA University, Institute of Health Sciences, İstanbul, Türkiye; ^3^ Department of Pharmacology, Acibadem MAA University, School of Pharmacy, Istanbul, Türkiye

**Keywords:** GPCR, orphan GPCRs, Alzhemier’s disease, Parkinson’s disease, Neurodegenaration

## Abstract

Neurodegenerative disorders represent a significant and growing health burden worldwide. Unfortunately, limited therapeutic options are currently available despite ongoing efforts. Over the past decades, research efforts have increasingly focused on understanding the molecular mechanisms underlying these devastating conditions. Orphan receptors, a class of receptors with no known endogenous ligands, emerge as promising druggable targets for diverse diseases. This review aims to direct attention to a subgroup of orphan GPCRs, in particular class A orphans that have roles in neurodegenerative disorders, including Alzheimer’s disease, Parkinson’s disease, Huntington’s disease, and Multiple sclerosis. We highlight the diverse roles orphan receptors play in regulating critical cellular processes such as synaptic transmission, neuronal survival and neuro-inflammation. Moreover, we discuss the therapeutic potential of targeting orphan receptors for the treatment of neurodegenerative disorders, emphasizing recent advances in drug discovery and preclinical studies. Finally, we outline future directions and challenges in orphan receptor research.

## Introduction

G protein-coupled receptors (GPCRs) also called seven transmembrane (7TM) receptors constitute the largest receptor family in the human protein atlas. GPCRs remain highly sought-after drug targets, owing to their ability to interact with numerous endogenous ligands. GPCRs are categorized into distinct classes based on sequence homology and functional similarities These are, Class A; rhodopsin-like receptors, Class B; secretin family, Class C; metabotropic glutamate receptors, Class D; fungal mating pheromone receptors, Class E; cAMP receptors, and Class F; frizzled (FZD) and smoothened (SMO) receptors (Lee et al., 2018). Among these families the largest is the Class A family, which also includes the class A orphan subgroup. GPCRs play a vital role in many physiological and pathological processes and mediate the signaling of nearly two-thirds of hormones and neurotransmitters (Spillantini et al., 1997). While GPCRs represent a vast array of potential therapeutic targets, there are still more than 140 GPCRs, notwithstanding the olfactory receptor family, for which the natural ligands are lacking. These so-called orphan receptors remain unexplored in terms of their endogenous ligands, molecular signaling pathways and functions (Sriram and Insel, 2018).

Despite their elusive nature, orphan GPCRs present an intriguing opportunity to unravel hidden molecular mechanisms and potential treatment avenues for many debilitating conditions. In this context, investigating the involvement of orphan receptors in the pathogenesis and progression of neurodegenerative diseases holds the promise of uncovering novel targets that could redefine the landscape of drug development and improve the lives of individuals affected by these disorders (Table 1). This exploration represents a dynamic and evolving area of research, poised to contribute significantly to the ongoing efforts to combat the complexities of neurodegenerative diseases.

**TABLE 1:** Orphan GPCR expression and ligands.

Orphan Receptors	Other Names	Expressed in	Gene	Signaling	Hypothetical Endogenous Agonists	Agonist
**GPR3**	• GPCR21	Brain	1p36.11	Gi	S1P (Uhlenbrock et al., 2002)	**Inverse Agonists**
	• GPCR3	Hippocampus Habenula		Gs (Freudzon et al., 2005)	DHS1P and DPI (Capaldi et al., 2018)	AF64394 (Jensen et al., 2014)
	• ACCA orphan receptor adenylate cyclase constitutive activator G protein-coupled receptor R4	Cortex Amygdala		ERK1/2 and Akt (Morales et al., 2018b)		Diphenyleneiodonium chloride (Ye et al., 2014)
	• Gpcr20			β-arrestin2 (Laun et al., 2019; Isawi et al., 2020)		
**GPR6**	• Sphingosine 1-phosphate receptor	Basal Ganglia	6q21	G_s_ (Uhlenbrock et al., 2002)	S1P (Ignatov et al., 2003b)	
	• GPR6	Striatopallidal neurons frontal cortex, retrosplenial cortex, hippocampus, amygdala, and hypothalamus		Gi/o, affecting Ca2+ mobilization (Laun et al., 2019)		
				β-arrestin (Laun et al., 2019)		
**GPR12**	• Gpcr01/20/21/12	Brain	13q12.13	Gαs (Tanaka et al., 2007)	S1P (Ignatov et al., 2003a)	
	• R334	Cerebral cortex		Gαi (Laun et al., 2019)		
		Hippocampus Striatum				
**GPR17**	• P2Y-like receptor	Oligodendrocyte precursor cells	2q14.3	Gαi	LTC4, LTD4 (Benned-Jensen and Rosenkilde, 2010)	
	• UDP/CysLT receptorR12 Uracil nucleotide/cysteinyl leukotriene receptor	Frontal cortex Striatum		Gβγ (Liu et al., 2021)	UDP-glucose,	
		Brain stem Medulla		Gαs	UDP-galactose,	
				Gαq	UDP (Ciana et al., 2006; Benned-Jensen and Rosenkilde, 2010)	
				β-arrestins increased Ca2+ flux (Chen et al., 2009)		
				ERK1/2		
**GPR18**	⁃ GPCRW		13q32.3	Gi/Go family, Gq/G11 family	N-arachidonoylglycine (Kohno et al., 2006)	**Agonists**
	⁃ NAGly receptor			Gαi/o		N-arachidonoylglycine
	⁃ N-arachidonoyol glycine receptor			PI3K/Akt-ERK1/2 (Matouk et al., 2017)		O-1602, abnormal cannabidiol
				MAPK (McHugh et al., 2010; McHugh et al., 2012) eNOS/NO (Fitzgerald et al., 2023)		Δ9-tetrahydrocannabinol
						Anandamide
						Arachidonylcyclopropylamide
						Cannabidiol
						AM251 (McHugh et al., 2012)
						PSB-KD107 (Schoeder et al., 2020)
**GPR37**	• EDNRBL/EDNRLB	Cerebellum Spleen	7q31.33	Gαi	The peptides prosaptide and prosaposin (Meyer et al., 2013)	**Agonists**
	• PAELR	Thymus Peripheral blood leukocytes		Gβγ	Regenerating islet-derived family member 4 (Reg4) (Rezgaoui et al., 2006; Meyer et al., 2013; Wang et al., 2016)	Neuropeptide head activator (Rezgaoui et al., 2006)
	• hET(B)R-LP	Lymph node		Gαs		
	• GPCR CNS1	Lung		Gαq		
	• Parkin-associated endothelin B-like receptor	Testis		β-arrestins increased Ca2+ flux		
	• Endothelin B receptor-like protein 1			MAPK		
				ERK1/2 (Yang et al., 2016)		
**GPR49**	• Lgr5	Glioblastoma stem cells	12q21.1	Gα i/o		**Agonists**
	• GPR49	Spinal cord		PLC		R-spondin-1
	• FEX	Motor neurons of brain stem		PKC		R-spondin-2
	• GPR67	Layer 5a and 6 neurons in cortex		β-arrestin (Snow et al., 1998)		R-spondin-3
	• Orphan G protein-coupled receptor HG38			MAPK/ERK PI3K/Akt pathways (Watson et al., 2018)		R-spondin-4 (Carmon et al., 2011)
	• Leucine-rich repeat-containing G protein-coupled receptor 5			G 12/13		
				Rho kinase Pathway (Kwon et al., 2013)		
**GPR50**	• MTNRL	Cortex	Xq28	MT receptors		
	• H9	Midbrain		GPR50/MT1 heterodimer is without G protein coupling (Levoye et al., 2006)		
	• Mel1c	Pons		ADAM17-Notch (Saha et al., 2020)		
	• Melatonin-related receptor	Amygdala Hippocampus except glial cells				
		Inhibitory interneurons				
**GPR52**		Prefrontal cortex Basal ganglia Striatonig	1q25.1	Gαs (Lin et al., 2020) PKA CREB ERK1/2 β-arrestin-2-dependent (Wang et al., 2020a; Hatzipantelis et al., 2020b)		**Agonists** Derivative 17 (Nakahata et al., 2018) Compound 7a: 3-[2-(3-Chloro-5-fluorobenzyl)-1-benzothiophen-7-yl]-N-(2-ethoxyethyl)benzamide (Setoh et al., 2014)
**GPR55**		central nerve tissues and cells	2q37.1	MAPK/ERK (PI3K)/Akt RhoA-dependent Ca2+ signaling NFAT (Henstridge et al., 2008) Gα12/13-RhoA-ROCK and Gαq-PLC-PKC(88) Gq-G11, mitogen-activated protein kinase 1, and calcium signaling (Henstridge et al., 2008; Andradas et al., 2011)	anandamide 2-arachidonoylglycerol 2-arachidonoylglycerolphosphoinositol lysophosphatidylinositol N-palmitoylethanolamine (Ryberg et al., 2007)	2-arachidonoylglycerol N-palmitoylethanolamine JWH015 O-1602 (Ryberg et al., 2007)*
**GPR78**		Brain Frontal cortex Putamen Thalamus Hypothalamus Amygdala Hippocampus Pons Medulla Midbrain	4p16.1	Gαs Gαq-Rho GTPase (Dong et al., 2016)	Teratocarcinoma-derived growth factor I (Cripto) DnaJ-like protein MTJ-1 α2-macroglobulin Kringle 5 Par-4 (Arap et al., 2004; Gonzalez-Gronow et al., 2009; Araujo et al., 2018)	**Agonists** BC71 and two peptides
**GPR83**	• GIR	Hippocampus Amygdala	11q21	Gαi/o		**Agonists**
	• GPR72	Prefrontal cortex		Gαq (Lueptow et al., 2018)		PEN (Uhlenbrock et al., 2002)
	• Glucocorticoid induced receptor	Various hypothalamic nuclei		MAPK (Müller et al., 2013)		Zn2+ (Müller et al., 2013)
	• G protein-coupled receptor 72					
	• JP05					
**GPR84**	• GPCR4	Microglial cells	12q13.13	Gαi/o (Suzuki et al., 2013b) MAPK/ERK (Wang et al., 2023)	Medium chain free fatty acids with carbon chain lengths of 9–14	**Agonists:** decanoic acid
	• Inflammation-related G-protein coupled receptor EX33				6-n-octylaminouracil (Suzuki et al., 2013a)	undecanoic acid
						lauric acid
						Embelin (orthosteric)
						PSB-16434 (orthosteric)
						ZQ-16 (orthosteric)
						6-nonylpyridine-2,4-diol (orthosteric)
						DL-175 (orthosteric)
						Allosteric modulator DIM (Agonist) (Wang et al., 2006; Suzuki et al., 2013a; Madar et al., 2021; Marsango et al., 2022)
**GPR85**	• Srep2	Hippocampal formation	7q31.1			New inverse agonists developed (Sakai et al., 2022)
	• SREB2/SREB	Olfactory bulb Cerebellum				
	• Super conserved receptor expressed in brain 2					
	• PKrCx1					
**GPR88**	• STRG	Striatum	1p21.2	Gαi/o (Ehrlich et al., 2017)		2-PCCA and RTI-13951–33 (Garisetti et al., 2023)
	• striatum-specific GPCR			β-arrestin (Laboute et al., 2020)		

*Those with multiple ligands have not been placed. Table has been updated from the https://www.guidetopharmacology.org/GRAC/FamilyDisplayForward?familyId=16#83

Genes: https://www.informatics.jax.org/marker/MGI:101908

**FIGURE 1:**
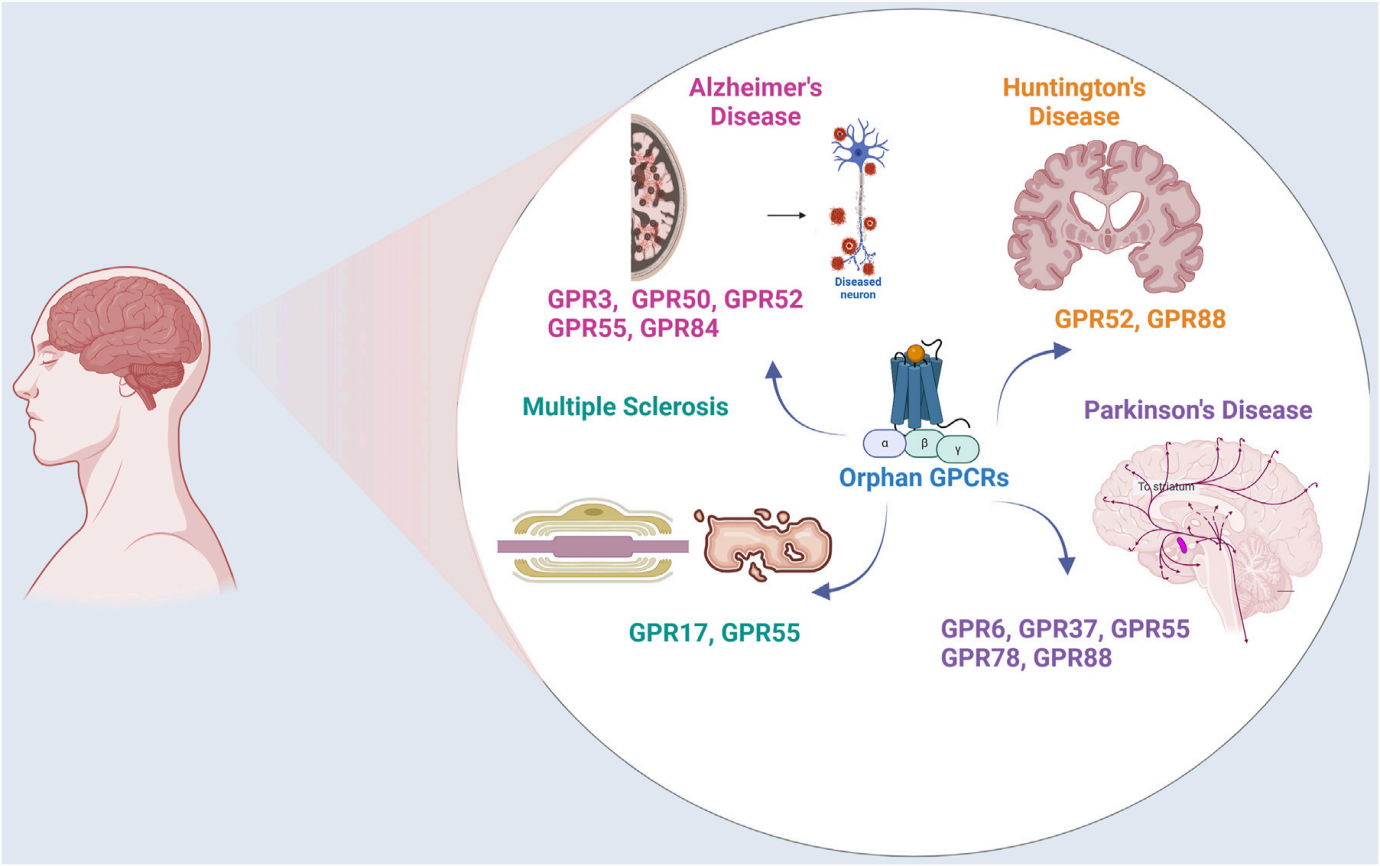
Orphan GPCRs in Neurodegenerative Disorders. The figure illustrates the expression and localization of orphan GPCRs in Parkinson’s Disease, Alzheimer’s Disease, Multiple Sclerosis, and Huntington Disease.

The research on orphan receptors has emerged as a promising area in chasing for novel drug targets, particularly for neurodegenerative disorders such as Parkinson’s disease (PD), Alzheimer’s disease (AD), Huntington’s disease (HD), and Multiple sclerosis (MS) (Figure 1). These disorders pose significant challenges, characterized by complex and multifaceted pathologies that currently lack comprehensive therapeutic solutions. Neurodegenerative disorders usually manifest as progressive decline in major functions such as cognition, motor functions and accompanying mood disorders depending on the anatomical region in the brain effected. For instance, PD is characterized by the gradual deterioration of dopaminergic neurons in the substantia nigra pars compacta, marked with the accumulation of intracellular protein inclusions known as Lewy bodies, composed of misfolded α-synuclein (α-syn) (Spillantini et al., 1997). The damage of PD is not restricted to the dopaminergic neurons in the substantia nigra but also expand to motor systems, the limbic system, medulla oblongata/pontine tegmentum and olfactory bulb and the autonomic centers, as inferred from the anticholinergic side effects of anti-Parkinson’s medications (Braak et al., 2004). AD is another age related neurodegenerative disease in which mitochondrial dysfunction, tau pathology, Aβ plaques and neurofibrillary tangles are deposited and lead to neuronal damage and cell death, primarily affecting memory and cognitive functions (Tiraboschi et al., 2004). The genetic neurodegenerative Huntington’s disease also causes a progressive breakdown of neurons, progressive tissue lost specifically in the caudate and cortical thinning related to distinct motor and cognitive phenotypes, affecting motor control, cognition, and behavior (Draganski and Bhatia, 2010). Another chronic inflammation-based pathology leads to MS, which targets the central nervous system (CNS). In MS the inflammation, demyelination, and neuronal damage (Korn, 2008) progresses into the axon injury/loss, which is followed by long-term physical and cognitive impairments (Springer, 2021).

Despite extensive research and progress in the de-orphanization of GPCRs, more than 1000 GPCRs are still classified as orphan receptors, without identified ligands and with unknown physiological functions. In this review, we draw attention to a subgroup of orphan GPCRs, in particular Class A orphans, including GPR3, GPR6, GPR12, GPR17, GPR18, GPR37, GPR49, GPR50, GPR52, GPR55, GPR78, GPR83, GPR84, GPR85, GPR88, that have links to neurodegenerative disorders.

We provide an overview of the proposed ‘’hypothetical’’ endogenous ligands, and designed ligands according to the identified structures and signaling pathways linked to these receptors (Table 1). We seek to integrate this knowledge with insights into the pathophysiology of neurodegenerative disorders, while also considering the relevant anatomical brain locations already associated and implicated in these neurodegenerative conditions.

### GPR3, GPR6, and GPR12

GPR3, GPR6 and GPR12 comprise a family of closely related orphan receptors that belong to the class A family of GPCRs (Laun et al., 2019). These orphan receptors display high constitutive activity and are capable of signaling through G protein-mediated and non-G protein-mediated mechanisms (Morales et al., 2018a; Laun et al., 2019). Three independent groups reported the molecular cloning of GPR3, GPR6 and GPR12 (Saeki et al., 1993; Eggerickx et al., 1995; Song et al., 1995). The genes encoding these receptors were located in the human chromosomal regions 1p36.1, 6q21 and 13q12, respectively. GPR3, GPR6 and GPR12 share over 60% sequence identity and common conserved motifs and structural features among them. GPR3 and GPR6 share common chromosomal positions with cannabinoid receptors, suggesting that they have a common ancestor (Fredriksson et al., 2003). Molecules targeting GPR3, GPR6 and GPR12 are of interest for therapeutic applications since they are implicated in several neurodegenerative diseases, including AD, PD, HD and MS. In addition to neurodegenerative disorders, these orphan receptors may impact other brain-related processes such as neuropathic pain, cocaine reinforcement or cell survival and proliferation (Morales et al., 2018a). Their high presence in the central nervous system and also their proposed roles in neurite outgrowth renders them valuable for the basic understanding of physiological processes and the underlying mechanisms of orphan GPCRs and possibly all GPCRs.

**GPR3**: GPR3 is extensively expressed in the brain, primarily in the hippocampus, habenula, cortex and amygdala (Eggerickx et al., 1995). Activation of GPR3 leads to an increase in adenylyl cyclase, which in turn augments the level of intracellular cyclic adenosine monophosphate (cAMP). It is known that cAMP plays significant roles in neurons including neurite outgrowth, axonal regeneration and axonal guidance. Tanaka et al. have demonstrated that neuronal expression of the GPR3 receptor enhances neurite outgrowth, and regulates the proliferation of cerebellar granule cell precursors (Tanaka et al., 2007; Tanaka et al., 2009). The same group of investigators have shown that GPR3 protects neurons from apoptosis via activation of ERK and AKT signaling (Tanaka et al., 2014). Conversely, adverse effects of GPR3 are implicated in the amyloid pathology observed in AD.

One of the pathological hallmarks of AD is the progressive accumulation of aggregates of amyloid peptides in the brain. The amyloid beta (Aβ) peptides are generated from the sequential breakdown of amyloid precursor protein (APP) by two peptides, the β- and γ-secretases. The β-secretases and γ-secretases play a fundamental role in APP proteolysis and Aβ generation. GPR3 has been identified to play a role in regulating the breakdown of APP, thereby modulating the progression of AD. In both neuronal cultures and animal models, GPR3 was shown to upregulate the γ-secretase activity and Aβ accumulation (Thathiah et al., 2009). In a subsequent study, the same researchers demonstrated that GPR3 messenger RNA (mRNA) levels were elevated in 18 post-mortem brain tissue of AD patients (Thathiah et al., 2013). The physiological consequence of loss of the GPR3 gene was investigated in four AD-mouse models by Huang et al., 2015 (Huang et al., 2015). These investigators observed that genetic deletion of GPR3 reduced amyloid pathology in all of the AD mouse models they studied. These studies suggest that lowering GPR3 activity may be beneficial in reducing amyloid pathology in AD.

A Lysophospholipid sphingosine-1-phosphate (S1P) has been suggested as an endogenous ligand of GPR3 in rats (Uhlenbrock et al., 2002). DHS1P and DPI are also potential endogenous ligands for GPR3 mentioned in the literature (Uhlenbrock et al., 2002; Capaldi et al., 2018). Furthermore, an inverse agonist, AF64394, has been proposed for GPR3 (Jensen et al., 2014; Kaushik and Sahi, 2017).

**GPR6:** GPR6 was initially described as S1P receptor (Morales et al., 2018a; Atanes et al., 2021). It is co-localized to dopamine D1 and D2 receptors, as are GPR52 and GPR88 (Rahman et al., 2022). GPR6 which is extensively expressed in striatopallidal neurons in the basal ganglia (Lobo et al., 2007; Komatsu et al., 2014) has ubiquitous functions and it induces an increase in cAMP levels when it is linked to stimulatory Gs protein. It plays a significant role in human instrumental learning in which the dopaminergic system has a critical role. In rodent cerebellar granule neurons, overexpression of GPR6 boosts neurite outgrowth (Tanaka et al., 2007; Laun et al., 2019). On the other hand, studies carried out in GPR6-knock-out mouse models suggest that GPR6 inhibition may provide benefits for PD. In GPR6-knock out mice, phosphorylation of dopamine and cAMP-regulated phosphoprotein of 32 kDa (DARPP-32) at threonine 34 increased significantly, while production of DARPP-32 in the striatum did not (Oeckl et al., 2014; Nishi and Shuto, 2017).

**GPR12:** GPR12 is phylogenetically related to the Cannabidiol Receptors (CB-1 and CB-2) (Wong et al., 2023). GPR12 is also a constitutively active receptor expressed mainly in the central nervous system. S1P (Eggerickx et al., 1995; Uhlenbrock et al., 2002; Martin et al., 2015) and sphingosine-phosphorylcholine (SPC) (Ignatov et al., 2003a; Allende et al., 2020) are potential endogenous ligands for GPR12. GPR 12 is expressed mainly in the central nervous system, in structures related to cognitive processes such as the cerebral cortex, the hippocampus and the striatum (Saeki et al., 1993). In mice, GPR12 is expressed in the area controlling emotion and metabolism (Ignatov et al., 2003a). Other functions ascribed to GPR12 include pain control, neurite outgrowth and regeneration (Allende et al., 2020). A study in rat pheochromocytoma PC12 cells demonstrated that GPR12 overexpression promotes neurite outgrowth by inducing differentiation of PC12 into neuron-like cells. This effect was accompanied by activation of ERK1/2 signaling (Lu et al., 2012). A report based on SNP microarray-based genome-wide association suggests a link between GPR12 and antipsychotic response to schizophrenia treatment (Zhao et al., 2022).

### GPR17

GPR17 is an orphan GPCR that is expressed in oligodendrocyte precursor cells (OPCs) and premature oligodendrocytes (Fumagalli et al., 2011). GPR17, a purinergic P2Y-like receptor, responds both to uracil nucleotides (UDP, UDP-glucose, UDP-galactose) and cysteinyl leukotrienes CysLTs, such as LTD4 and LTC4. These endogenous ligands are released extracellularly at sites of neuroinflammation, where GPR17 is elevated (Fumagalli et al., 2011). The expression of GPR17 increases during damage to nerve cells. Furthermore, it takes part both in the process of inducing damage and also in the local repair of the damaged myelin sheath. Thus, GPR17 is an attractive target for MS.

The GPR17 gene was first isolated in 1996 and characterized for the first time in 2006 (Raport et al., 1996; Ciana et al., 2006). Phylogenetically, GPR17 is closely related to the purine subfamily and cysteinyl leukotriene receptors CysLT1 and CysLT2 (Fumagalli et al., 2016). It has been classified into the rhodopsin-like family, together with the purinergic P2Y receptor. In humans, the gene for GPR17 is located on chromosome 2q21. GPR17 receptors are present in neurons and some parenchymal quiescent OPCs. GPR17 is one of the key proteins expressed in human adult neuroprogenitor cells and participates in neuronal repair. GPR17 receptors are found in abundance in the nervous system, including the frontal cortex, striatum, brain stem and medulla (Ceruti et al., 2011). In addition, it is expressed in organs that undergo ischemic injury, including the brain, kidney and heart (Ciana et al., 2006; Ceruti et al., 2011; Dziedzic et al., 2020).

The level of GPR17 receptors is increased in oligodendrocyte lineage cells during the differentiation of OPCs into premature oligodendrocytes (Alavi et al., 2018). Recent reports indicate that GPR17 receptors play a role in both demyelination and remyelination processes in the central nervous system (CNS) (Dziedzic et al., 2020). These receptors seem to contribute to the death of neurons in sites of inflammation and also cause nerve tissue repair. Myelin sheath destruction and axonal injury are among the hallmarks of MS. The presence of oligodendrocytes and intact myelin sheath are essential for the proper functioning of neurons. Thus, they can serve as a sensor for local damage to the myelin sheath and as a potential marker of the neurodegenerative process in MS. In an *in vivo* mouse model of MS that presents clinical and pathological similarities to human MS, a highly selective GPR17 agonist delayed the onset of encephalomyelitis (Parravicini et al., 2020).

### GPR18

GPR18 was first cloned by Gantz et al., in 1997. In humans, GPR18 is abundantly expressed in the spleen, thymus, peripheral blood leukocytes, lymph node, cerebellum, lung and testis, among others (Gantz et al., 1997; Vassilatis et al., 2003). GPR18 is also expressed in several immune cell types where it is involved in different biological functions. It shares low sequence homology with the cannabinoid receptors CB-1R and CB-2R and displays moderate identity with the putative cannabinoid receptor GPR55 (Morales et al., 2020).

GPR18 regulates polymorphonuclear cell infiltration and protects organs from acute immune responses (Reyes-Resina et al., 2018). The interest in GPR18 lies in its ability to recognize cannabinoid ligands and its propensity to heteromize with CBRs. This suggests that GPR18 and its heteromers may be attractive targets for neurodegenerative disorders.

The therapeutic potential of GPR18 has been shown through *in vitro* and animal model studies. It has been shown that GPR18 can interact with the CB-2R in activating microglia of the AD model. Two different compounds have been proposed as putative ligands for GPR18; however, due to insufficient *in vivo* data, GPR18 is still grouped under class A orphan GPCRs. (for an extensive review on GPR18, see Morales et al., 2020) (Morales et al., 2020).

### GPR37

Among orphan receptors with therapeutic potential for the treatment of neurodegenerative diseases, GPR37 is of particular interest since it is extensively expressed in the brain and the central nervous system and because it is related to the dopaminergic system and brain myelination. GPR37 is recognized as the parkin-associated -endothelin receptor-like receptor (Pael receptor) since it was originally identified as a substrate of parkin (Imai et al., 2000; Imai et al., 2001). Parkin is an E3 ubiquitin ligase encoded by the PARK2 gene involved in ubiquitination and proteasome-mediated degradation of misfolded proteins (Imai et al., 2001). Mutations in the PARK2 gene are the most common cause of autosomal recessive juvenile parkinsonism (AR-JP) (Kitada et al., 1998). An insoluble form of GPR37 was reported to accumulate in the brains of AR-JP patients. It is worth mentioning that modulation of GPR37 signaling is implicated in other diseases such as bipolar and major depression disorders, autism and epilepsy.

The GPR37 gene was first discovered in humans and localized to chromosome 7 (7q3l) as encoding for 7TM 613 amino acid-protein (Marazziti et al., 1998). Immunohistochemical mapping of GPR37 protein levels in mouse brain showed that the receptor is widely expressed in oligodendrocytes, whereas neuronal expression is mainly limited to the nigrostriatal dopaminergic system and hippocampus (Imai et al., 2001).

Although its physiological relevance remains to be elucidated, GPR37 may be an attractive target for PD, since it interacts with the D2R, the 5-HT4R and also with the adenosine A_2A_ receptor, A_2A_R. In PD, GPR37 acts as an A_2A_R inhibitor via receptor oligomerization. Recent studies suggest that GPR37 has a bidirectional role in PD pathogenesis. While its physiological role seems to be neuroprotective, it can misfold and aggregate intracellularly, ultimately leading to cell death (Leinartaité and Svenningsson, 2017).

Thus far, three different molecules, the head activator (HA), prosaposin (PSAP) and regenerating islet-derived family member 4 (Reg4) have been suggested to signal via GPR37, (Rezgaoui et al., 2006; Meyer et al., 2013; Wang et al., 2016), but currently GPR37 remains as a de-orphanized GPCR. Since GPR37 toxically accumulates in AR-JP, Morato et al. have explored the possibility of ecto-GPR37 as a potential biomarker for PD. Briefly, the presence of peptides from the N-terminus cleaved domain of GPR37 (i.e., ecto-GPR37) in human cerebrospinal fluid (CSF) samples of control subjects, PD patients and AD patients were identified by LC-MS analysis and quantified by an in-house ELISA method (Morató et al., 2021). The authors reported that significantly higher levels of ecto-GPR37 were detectable in the CSF of PD patients, but not in AD patients. Therefore, these authors suggest that ecto-GPR37 may be a promising potential biomarker for PD.

### GPR49

GPR49, also known as Lgr5, that plays a critical role in various cancers, including basal cell carcinoma, head and neck squamous cell carcinoma, oral squamous cell carcinoma, and hepatocellular carcinoma (Yamamoto et al., 2003; Tanese et al., 2008; Major et al., 2013). Recent studies show that LCR5 is also expressed in neuronal stem cells such as glioblastoma stem cells and is associated with neuronal differentiation and maturation (Mao et al., 2013). In addition, LGR5 is abundantly expressed in spinal cord, motor neurons in brain stem, and neurons in Layer 5a and 6 in cortex, thereby LGR5 might be involved in the development of projection neuron in CNS (Song et al., 2015). Based on its expression in motor neurons and cortex, GPR49 may play a crucial role in the development of neurodegenerative diseases, although no studies have identified a link between this receptor and these disorders.

### GPR50

GPR50 is widely distributed in many brain region such as cortex, midbrain, pons, amygdala and hippocampus except glial cells (Grunewald et al., 2012). In addition, it is also expressed in the inhibitory interneurons, These data suggest that GPR50 might modulate the excitability of neurons and regulate synaptic plasticity and cognitive function (Li et al., 2020). Furthermore, there is a growing evidence showing that GPR50 may be involved in the hypothalamus–pituitary–adrenal (HPA) axis and the glucocorticoid receptor (GR) signaling, leptin signaling, adaptive, thermogenesis, torpor and neuronal differentiation (Bechtold et al., 2012; Khan and He, 2017).

Although there is a potential link between GPR50 and psychiatric conditions and given the overlap between psychiatric and neurological disorders, recent findings have addressed the role of GPR50 in neurodegenerative diseases. Chen et al. (2019) have demonstrated a significant link between GPR50 hypomethylation and AD in males, suggesting a potential role for GPR50 in the development or progression of AD (Chen et al., 2019).

Moreover, GPR50, previously known as melatonin-related receptor, was cloned from the human pituitary and recognized as a member of the melatonin receptor subfamily and showed high amino acid similarities (45%) with MT1 and MT2 (Reppert et al., 1996). Zlotos et al. (2014) suggested the potential heterodimerization of melatonin receptor subtypes, including MT1 and MT2, with GPR50, which might affect melatonin receptor function (Zlotos et al., 2014). Changes in these receptors' expression patterns may contribute to the development and progression of the disease, pointing to a possible link between GPR50-related melatonin signaling pathways and neurodegenerative diseases like AD. Although the importance of GPR50 and ligand interactions has been established for neurodegenerative diseases, further research is needed to clarify downstream signaling pathways.

### GPR52

GPR52 is predominantly expressed in the brain, particularly in regions associated with symptoms of neuropsychiatric disorders and Huntington’s disease (Komatsu et al., 2014; Nishiyama et al., 2017; Hatzipantelis et al., 2020a; Wang P. et al., 2020; Ma et al., 2020). Along with GPR6 and GPR8, GPR52 shows promise as a therapeutic psychiatric receptor, especially due to its association with dopamine receptors in the basal ganglia (Rahman et al., 2023).

In light of its involvement in cAMP signaling pathways and potential effects on physiological functions, the expression and signaling cascade of the orphan receptor GPR52 has drawn attention in recent years. GPR52 has been found to co-localize with D1 receptors in the prefrontal cortex and with D2 receptors in the basal ganglia, indicating its involvement in dopaminergic transmission in these regions (Komatsu et al., 2014; Constantinof et al., 2019). In addition, the expression profiles in the prefrontal cortex overlap with D1 dopamine receptors, suggesting a potential influence on locomotor activity through the activation of DRD1 and NMDA receptors via cAMP accumulation (Hatzipantelis et al., 2020a). Moreover, it has been suggested that GPR52’s activation of ERK1/2 signaling and the recruitment of β-arrestins in frontal cortical neurons are mechanisms that require further investigation (Woo et al., 2020).

GPR52 signaling via cAMP has been implicated in opposing D2 signaling in the striatum while stimulating D1/NMDA function in the frontal cortex (Russell et al., 2021). However, the effectiveness of GPR52 agonism in modulating D2/3 receptor signaling outside of the striatum may be limited by lower expression levels (Rahman et al., 2022). Moreover, GPR52-expressing neurons in the habenular nucleus have been suggested to provide negative compensatory signals to dopaminergic neurons in the midbrain. GPR52 has also been linked glutamatergic transmission in addition to the modulation of dopaminergic transmission, further emphasizing its role in cognitive and emotional processes (Komatsu et al., 2014).

Furthermore, the identification of GPR52 selective antagonists through high-throughput screening and studies of the structure-activity connection presents novel possibilities for therapeutic approaches for diseases such as Huntington’s disease (Komatsu, 2021).

Reducing GPR52 or using antagonist can result in a decrease in soluble mutant Huntingtin (mHTT) protein levels, thereby improving HD-like phenotypes (Yao et al., 2015). This effect is linked to the modulation of mHTT levels through the inhibition of GPR52 function (Wu et al., 2023). Additionally, research has shown that GPR52 plays a role in rescuing behavioral phenotypes in HD mouse models, indicating its potential as a therapeutic target for the disease (Stott et al., 2021).

An understanding of the complex relationship between GPR52 and other receptors like dopamine D2 will help to develop novel treatment strategies that could address the complex pathophysiology of conditions like PD.

### GPR55

G-protein coupled receptor 55 (GPR55) is widely expressed in the central nerve tissues and cells, and plays a role in controlling oxidative and inflammatory cell homeostasis (Apweiler et al., 2021). GPR55 interacts with two cannabinoid receptors (CB1/CB2). GPR55 forms heteromer structure with CB1 and CB2 receptors like other orphan receptors (GPR3/GPR6/GPR12/GPR18), or PPARγ, subsequently leading to complex interactions that can either inhibit or enhance GPR55-mediated signaling (Apweiler et al., 2021; Perez-Olives et al., 2021).

GPR55 plays a crucial role in various cellular processes such as cell proliferation, migration, survival, and tumorigenesis in various cancer cell lines (Hasenoehrl et al., 2019; Akimov et al., 2023). It triggers a cascade of signaling events by interacting with different receptors or ligands, leading to diverse outcomes in different cell types (Balenga et al., 2014; Shi et al., 2017; Hill et al., 2019). Additionally, GPR55 has been implicated in modulating neurotransmitter release at central synapses, further highlighting its diverse functions (Sylantyev et al., 2013).

Furthermore, it has been demonstrated that GPR55 stimulates the extracellular signal-regulated kinase (ERK) cascade, which in turn stimulates the growth of cancer cells (Andradas et al., 2011; Mangini et al., 2017). GPR55 has been shown to promote cancer cell proliferation via the extracellular signal-regulated kinase (ERK) cascade (Andradas et al., 2011; Mangini et al., 2017). The cell type-dependent RhoA activation reported upon activation of GPR55 in different studies indicate to cell type-specific downstream signaling cascade (Liu et al., 2016).

Numerous physiological processes and diseases, such as neuropathic pain, cancer, metabolic diseases, inflammatory pain, bone growth, and neurological disorders have been linked to GPR55 (Kotsikorou et al., 2013). While its role in diseases like obesity, diabetes, osteoporosis, and cancer has been studied, its involvement in CNS disorders such as depression, AD, and PD remains poorly understood (Apweiler et al., 2021). It has been implicated in various physiological processes and diseases, including neuroinflammation and neurodegenerative conditions like Multiple sclerosis (MS) (Saliba et al., 2018).

Very recent studies have delineated the association between GPR55 and cannabioid receptors and cannabidiol (CBD), a cannabinoid compound. CBD has been found to cause vasorelaxation through CB1 activation and has been implicated in modulating seizures through interactions with CB1, CB2, GPR18, GPR55, and other receptors (Stanley et al., 2015; Longoria et al., 2022). Additionally, CBD has been used in clinical practice for conditions like spasticity in MS and childhood epilepsy (Hind et al., 2016; Golub and Reddy, 2021). A number of studies demonstrate that the GPR55, PPARγ, and TRPV channels signaling pathways are linked to the anti-inflammatory effects of CBD (Lötsch et al., 2018). Since inflammation is one of the hallmarks of neurodegenerative diseases, targeting GPR55 might be a novel therapeutic approach for the treatment of neurodegenerative diseases like PD, AD, and MS (Saliba et al., 2018).

Recent evidence has pointed out that single nucleotide polymorphisms (SNPs) in GPR55 are linked to AD progression, suggesting a role in the disease (Mori-Fegan et al., 2023). In AD mouse models, activation of GPR55 has been shown to reduce synaptic dysfunction, oxidative stress, neuroinflammation, and cognitive impairment (Xiang et al., 2022). Furthermore, research indicates that endocannabinoid-related receptors, such as GPR55, are expressed more abundantly in mice models of AD, indicating that these receptors have a role in the disease (Medina-Vera et al., 2023). GPR55 is also expressed on microglia cells which are known to be essential for neuroinflammation. GPR55 antagonists have also been shown to have anti-neuroinflammatory properties in microglial cells, suggesting a possible treatment path for neurological disorders characterized by neuroinflammation (Saliba et al., 2018).

Activation of GPR55 has been observed to mitigate cognitive impairment, oxidative stress, neuroinflammation, and synaptic dysfunction in AD mouse models (Xiang et al., 2022). The GPR55 agonist, O-1602, has displayed a potential in ameliorating cognitive impairment, neuroinflammation, oxidative stress, and apoptosis induced by lipopolysaccharide in mice, suggesting a neuroprotective role (Wang XS. et al., 2020).

In addition to AD, GPR55 has been implicated in PD. Recent studies have shown high expression of GPR55 in the striatum and in the external globus pallidus, indicating a potential link between GPR55 activity and motor dysfunction in PD (Patricio et al., 2022; Wong et al., 2023). GPR55 and CB1 heteromers have also shown significant neuroprotection against parkinsonism-inducing toxins, as in AD (Cooray et al., 2020). Additionally, the expression of heteromers consisting of GPR55 and CB1/CB2 receptors in the striatum has been evaluated in parkinsonian macaques, highlighting a correlation between Parkinsonism and altered expression of these heteromers (Basile and Mazzon, 2022). The use of GPR55 as a therapeutic target for managing motor deficits in PD has been proposed, with research focusing on the effects of GPR55 selective ligands in PD rat models (Fatemi et al., 2021; Sánchez-Zavaleta et al., 2023). The therapeutic potential of GPR55 has been also explored in PD, with studies indicating that GPR55 activation may reduce circuit dysfunction in PD-related afferent systems, making it a promising approach for treating disease-related motor dysfunction (Hewer et al., 2023).

In experimental autoimmune encephalomyelitis models of MS, the genetic background has been found to influence the effects of gene knockout, particularly of GPR55 and CB2 receptors, on disease severity (Nouh et al., 2023). Specifically, GPR55 has been associated with pro-inflammatory roles in mouse models of gastrointestinal inflammation and MS (Kurano et al., 2021; Nouh et al., 2023).

### GPR78

GPR78 is closely analogous to the GPR26 gene and is exclusively identified in the placenta and pituitary glands of humans. There were no mRNA transcripts detected in other central nervous system regions, including the frontal cortex, putamen, thalamus, hypothalamus, amygdala, hippocampus, pons, medulla, and midbrain (Lee et al., 2001). GPR78 is generally expressed in endoplasmic reticulum and inactivates ER stress sensors ATF-6, PERK and IRE1 (Araujo et al., 2018). *In vitro*, GPR78 is shown to increase intracellular cAMP (Jones et al., 2007). Acting as a regulator within the phosphoinositide 3-kinase (PI3K)–protein kinase B (AKT) signaling network, it exerts varied downstream effects on the proliferation, survival, metastasis, and chemoresistance of cancer cells. BC71 and two peptides have been developed for the GPR78 receptors (Arap et al., 2004; Gonzalez-Gronow et al., 2009; Araujo et al., 2018). Since GPR78 is expressed in the basal ganglia, it may be also be involved in the pathophysiology of PD.

### GPR83

GPR83, also known as JP05, GIR, and GPR72, is a GPCR initially identified in thymoma as a glucocorticoid-induced receptor. It is extensively present in CD4^+^CD25^+^ regulatory T (Treg) cells and the central nervous system, particularly in brain regions like the hippocampus, amygdala, prefrontal cortex, and various hypothalamic nuclei. GPR83 is implicated in stress-associated physiology and may play significant roles in learning and memory, reward, emotional behaviors, and stress regulation. GPR83-deficient mice showed delayed spatial learning acquisition and an increased preference for sucrose (Li DY. et al., 2013; Li J. et al., 2013). GPR83 and GPR171 signaling pathways in brain regions control feeding and reward behaviors. Recently, there has been discussion concerning the de-orphanization of GPR83, attributed to its discovery of binding with bigLEN or PEN, known to regulate feeding behavior (Gomes et al., 2016). FAM237A and FAM237B are the ligands which are shown to activate GPR83, and the latter activate GPR83 through the Gαq signaling pathway (Sallee et al., 2020). Although a direct correlation between GPR83 and neurodegenerative disorders has not been demonstrated yet, its involvement in learning and memory may have an impact for AD research.

### GPR84

GPR84, a Gi-coupled GPCR, has been suggested to recognize endogenous medium-chain fatty acids (MCFAs) (Fredriksson et al., 2003; Foord et al., 2005). Similar to GPR83, GPR84 also takes part in immune defense through microglia, which are essential in immune defense of the CNS and its diseases (Simard et al., 2006). In addition to modulating the microglial cells, GPR84 modulates the production of interleukin-4 (IL-4) by T lymphocytes, as well (Wittenberger et al., 2001; Yousefi et al., 2001; Venkataraman and Kuo, 2005).

In an experimental study, the gene of GPR84 is upregulated in microglial cells within the brains of APP/PS1 transgenic mice, a model for AD. The increased GPR84 activity correlates with faster cognitive decline and a decrease in the number of microglia, particularly around areas with amyloid plaques. Interestingly, the absence of GPR84 does not impact the formation of plaques or the hippocampal neurogenesis, but leads to β-amyloid-induced microgliosis and therefore contributes to the β-amyloid-induced dendritic degeneration (Audoy-Rémus et al., 2015). A recent study of human data using machine learning methods, where human samples from the entorhinal cortex bearing neurofibrillary tangles or none were examined, showed that among the other genes, GPR84 gene is differentially expressed. This suggests GPR84 has a potential to be a marker for AD (Madar et al., 2021). 6-n-octylaminouracil and 9–14 carbon chain fatty acids have been proposed as endogenous ligands (Wang et al., 2006; Suzuki et al., 2013a). Decanoic acid, lauric acid, embelin, PSB-16434, ZQ-16, 6-nonylpyridine-2,4-diol, DL-175 are orthosteric and DIM is an allosteric agonist (Wang et al., 2006; Suzuki et al., 2013a; Southern et al., 2013; Marsango et al., 2022).

### GPR85

GPR85 previously named as, Super conserved receptor expressed in brain-2, SREB2, has been associated with the brain, and has been linked to autism spectrum disorder and schizophrenia, so far. It has a neuroectodermal origin and is highly expressed in the mouse cerebral cortex and human adults. Its expression is increased with development and neuronal differentiation (Hellebrand et al., 2001). In rat, the expression of the gpr85 gene was found to be declined gradually after birth and became undetectable by postnatal day 18, but its weak expression was observed in the adult hippocampal formation, olfactory bulb, and cerebellum (Jeon et al., 2002). mRNA profiling across the species of adult human, monkey, and rat forebrains, SREB2 mRNA were detected in the hippocampal dentate gyrus, hippocampal formation, olfactory system, and supraoptic and paraventricular nuclei (Matsumoto et al., 2005).

Few studies have explored the association of GPR85 with brain disorders. However, its expression holds potential as a target for conditions like schizophrenia or epilepsy. Overexpression in transgenic mice resulted in decreased social interaction, abnormal sensorimotor gating, and impaired memory. Additionally, GPR85 expression increased in the adult hippocampal formation, piriform cortex, and amygdaloid complex following treatment with kainic acid, which induces convulsive epilepsy (Jeon et al., 2002; Matsumoto et al., 2008).

Studies designing novel ligands for GPR85 continues and so far, a new inverse agonist has been identified (Sakai et al., 2022). The association of GPR85 with learning and memory suggests a potential link to AD. GPR85 continues to be investigated with specific ligands and antagonists.

### GPR88

GPR88 exhibits widespread expression in the spleen, liver, and brain. It is conserved between humans and mice and is mapped to the 1p21.3 chromosomes in humans and 3G1 in mice. Initially characterized as a receptor specific to the striatum, GPR88 plays a role in various physiological processes within the central nervous system (Mizushima et al., 2000).

As it is connected to the striatum, it is no surprise that GPR88 has been extensively investigated with animal models of PD research. GPR88 is mainly expressed in the striatum of rodents, humans and is specifically associated with movement disorders (Ye et al., 2019). Knocking down Gpr88 negatively affected the expression of DARPP-32, a key protein in medium spiny neurons controlling dopamine reception. Gpr88 knockout mice showed increased spontaneous locomotion, drug-induced catalepsy sensitivity, and motor incoordination, suggesting GPR88’s role in motor function. While direct links between Gpr88 mutations and human PD are lacking, sporadic chorea cases in humans have been associated with mutations in GPR88 (Ye et al., 2019).

One of the most problematic issue in the management of PD is L-DOPA mediated tardive dyskinesia due to the long term use of dopaminergic agents. GPR88 proteins seem to be promising targets for the mitigation of dyskinesia. For instance, Gpr88 knockdown seem to prevent the onset of dyskinesia (Mantas et al., 2020). In this study while Gpr88 knockout mice exhibited less involuntary movements, less serotonin displacement and reduced tacrine-induced PD-like tremor and spontaneous locomotion (Mantas et al., 2020). An association between HD, an autosomal dominant condition which emerges around midlife, and GPR88 has also been proposed. (Rocher et al., 2016). mHTT, that affects striatal medium spiny neurons (MSNs) sustain their functionality over several decades (Rocher et al., 2016). In an *in vivo* study with a mice model of HD, BACHD, in which there is high expression levels of neuropathogenic, full length mutant huntingtin (fl-mHTT) genes, lower expression of GPR88 has been found in the striatum, that is accompanied by hyperexcitability, increased amplitude of AMPA receptor-mediated synaptic and a decline in spine density (Rocher et al., 2016).

Similarly, in a Gpr88-inactivated lentiviral-mediated knock-down striatal 6-OHDA rat model, a specifically designated microRNA (miR) (KD-Gpr88) reduced acute amphetamine-induced turning behavior and normalized striatal Gad67 and proenkephalin expression, indicating to an association with the severity of L-DOPA induced dyskinesia (Ingallinesi et al., 2019). In a further study of the same group, using medial forebrain bundle injections in an early Parkinson (6-OHDA)_ model, lentiviral-delivery of the specific microRNA to knock down GPR88 seemed to mitigate mood, motivation, and cognition alterations by modulating the regulator of G-protein signaling 4 and the truncated splice variant of the FosB transcription factor (Galet et al., 2021). GPR88 primarily couples to Gi/o proteins (Jin et al., 2018) and its known agonists are 2-PCCA and RTI-13951-33 (Garisetti et al., 2023). In summary, GPR84 may be a promising target in PD and HD in the future.

## Discussion

GPCRs are primary targets for drug development. Many drugs used today are the results of sustained research, stimulated by recent findings of additional signaling pathways. Orphan GPCRs hold the potential of being novel therapeutic targets for disorders that currently have no radical therapies. However, the allure of orphan GPCRs comes with a caveat. GPCRs, in general, are intricately linked to diverse signaling pathways, making them less than ideal drug targets. The association with multiple pathways raises concerns about potential side effects, already encountered with existing GPCR targets. Nevertheless, research on orphan GPCRs is expected to enhance our comprehension of their specific physiological and neuropathological functions in the years ahead. Orphan GPCRs have been associated with various physiological processes, including neuromodulation (Civelli, 2012), circadian behavior regulation (Doi et al., 2016), and immune response modulation that makes them this promising targets with the disorders that have these bases. Orphan GPCRs are also implicated in immune responses, with some receptors regulating key immune cells through metabolite signaling (Husted et al., 2017). Moreover, orphan GPCRs have shown a preference for associating with lipid and lipid-like molecules, suggesting a potential role in lipid metabolism and signaling pathways (Im, 2004; Wei et al., 2017; Jobe and Vijayan, 2024).

Sphingosine-1-phosphates (S1Ps) are signaling lipids which act on the S1PR family of cognate GPCRs and have been shown to modulate neuroinflammation, a process known to be involved in both neurodegenerative and cerebrovascular diseases (Chua et al., 2020). S1P, as an agonist on GPR3, GPR6, GPR12, may be a promising target in Alzheimer’s Disease (Kunkel et al., 2013). Despite the promising roles of GPR3 (Huang et al., 2015; Capaldi et al., 2018), GPR6, and GPR12 in neurodegenerative diseases, including AD, further research is required to fully elucidate their mechanisms of action and validate them as viable therapeutic targets. Sphingosine 1-phosphate (S1P) also plays a crucial role in inflammation, particularly in the context of MS. For example, Fingolimod (FTY720), an S1P receptor modulator, has been approved as an oral treatment for relapsing forms of MS, highlighting the relevance of S1P in MS treatment (Choi et al., 2010). Therefore, agonists on GPR3, GPR6, GPR12 can be considered as candidates for MS research.

Due to the heterogeneous expression of some orphan GPRCs such as; GPR3, GPR6, GPR18 and GPR55 with cannabinoid receptors of CB1 and CB2, the modulatory effects of CB receptors seem to be also intrinsically regulated by these specific orphan receptors. CB2 receptors, in particular, have been shown to have neuroprotective effects in conditions like HD by attenuating microglial activation and preventing neurodegeneration (Rajesh et al., 2007). CB1 receptors have been shown to provide neuroprotection through the inhibition of excitotoxicity and oxidative stress, as evidenced in animal models of some neurodegenerative diseases such as MS (Maresz et al., 2005; Gowran et al., 2010). The endocannabinoid system, involving CB1 and CB2 receptors, their ligands, and associated enzymes, acts as a key modulatory system influencing various pathological processes in neurodegenerative disorders (Jhaveri et al., 2007).

Furthermore, many orphan GPCRs, such as GPR83, GPR84 and GPR85 are expressed in various tissues, including immune system cells, Tregs, monocytes, macrophages, microglia and in the different brain regions (Wei et al., 2017). GPR83 has been linked to the regulation of stress, mood, reward-related behaviors, and immune function (Lueptow et al., 2018). GPR84 has been proposed to be involved in microglial motility after neuronal injury, suggesting a potential role in neuroprotection (Wei et al., 2017). Overall, these results indicate that orphan GPCRs are versatile receptors with implications in both immune responses and neuronal functions. Their involvement in inflammation and immune cell regulation, and along with their proposed neuroprotective roles make them promising targets for therapeutic interventions in conditions involving immune dysregulation and neuroinflammation.

Furthermore, current progress in GPCR structural biology, virtual libraries, molecular modelling and the use of cryo-EM for structure elucidation, has made a significant impact on overcoming different of obstacles in identifying orphan GPCRs ligands. These studies that emphasize the heterodimer or oligomeric structure of orphan GPCRs are critical for their function and signaling.

In conclusion, orphan GPCRs are widely expressed in the CNS and are involved in a wide range of physiological effects. Understanding the interactions of orphan receptors with other GPCRs such as cannabinoid receptors, dopamine receptors and melatonin receptors and their propensity to form heteromers will provide insights into the complex structural and functional mechanisms underlying neurodegenerative disease (Figure 1). Deciphering the ligands for orphan GPCRs and understanding their structure and signaling mechanism will facilitate identification of small molecules targeted for the therapy of neurodegenerative diseases.
